# Natural Killer Cells from Patients with Chronic Rhinosinusitis Have Impaired Effector Functions

**DOI:** 10.1371/journal.pone.0077177

**Published:** 2013-10-18

**Authors:** Ji Heui Kim, Gye Eun Kim, Gye Song Cho, Hyung-Joon Kwon, Chul Hyun Joo, Hun Sik Kim, Yong Ju Jang

**Affiliations:** 1 Department of Otolaryngology, Asan Medical Center, University of Ulsan College of Medicine, Seoul, Republic of Korea; 2 Department of Medicine, Graduate School, University of Ulsan College of Medicine, Seoul, Republic of Korea; 3 Department of Microbiology, University of Ulsan College of Medicine, Seoul, Republic of Korea; 4 Cellular Dysfunction Research Center, University of Ulsan College of Medicine, Seoul, Republic of Korea; Centre de Recherche Public de la Santé (CRP-Santé), Luxembourg

## Abstract

Natural killer (NK) cells are multicompetent lymphocytes of the innate immune system that play a central role in host defense and immune regulation. Although increasing evidence suggests that innate immunity plays a key role in the pathogenesis of chronic rhinosinusitis (CRS), the role of NK cells in CRS has been poorly studied. This study aimed to characterize the peripheral blood NK cells from patients with CRS, and to compare the functions of these cells with those from non-CRS controls. The correlation between NK cell functional activity and prognosis was also assessed. Eighteen CRS patients and 19 healthy non-CRS controls were included. The patients with CRS were classified into two subgroups, namely a treatment-responsive group and recalcitrant group. NK cell degranulation was determined by measuring the cell surface expression of CD107a against 721.221 and K562 cells. Intracytoplasmic cytokine production was determined by flow cytometry. Compared to the controls, the NK cells of CRS group had an impaired ability to degranulate and to produce cytokines such as IFN-γ and TNF-α. The recalcitrant subgroup showed the most severe defects in NK cell effector functions. Moreover, the decreased NK cell functions in patients with CRS were associated with poor prognostic factors such as concomitant asthma and peripheral blood eosinophilia. NK cells, which were originally named for their ability to mediate spontaneous cytotoxicity towards diseased cells including infected cells, may play an important role in regulating the inflammatory process in CRS pathogenesis.

## Introduction

Chronic rhinosinusitis (CRS) is an inflammatory disorder that involves the mucosa of the nose and paranasal sinuses [1]. Despite its high prevalence and its significant impact on health, CRS pathophysiology remains incompletely understood. Microbial organisms have been implicated as the inflammatory stimuli; moreover, defects in immune functions may contribute to the chronic inflammatory state [2]–[4]. Previously, CRS was believed to be a disorder of the adaptive immune system, including the lymphocytes and their associated cytokines [5]; however, given the increasing appreciation that adaptive immune responses are secondary to changes in innate immunity [6], rhinological research has recently begun to focus on possible failures in the innate immune system [7].

Natural killer (NK) cells are cytotoxic lymphocytes that constitute a major component of the innate immune system that prevents microbial infections [8]. Although the role of NK cells in CRS has not yet been investigated, several studies have shown that NK cells play a key role in preventing chronic inflammatory lung disease: it appears that the ability of NK cells to protect from infection may limit the inflammation and the consequent fibrosis [9]. Notably, NK cell function is impaired in chronic infections such as pulmonary tuberculosis and in chronic inflammatory disorders of the airways such as chronic obstructive pulmonary disease (COPD) [10]–[13]. Besides their role in controlling infections, NK cells can regulate the multitude of adaptive immune responses in allergic airway diseases such as asthma and allergic rhinitis [14]–[17]. Moreover, experimental studies using murine models of asthma have shown that NK cells may participate in the regulation of eosinophilic inflammation during sensitization and ongoing inflammation [18], [19]. Notably, eosinophilic inflammation is considered to be a major pathological hallmark of CRS [20], and eosinophilic CRS tends to associate with asthma and elevated eosinophil counts in the peripheral blood [21]. Eosinophilic CRS, especially with concomitant asthma, are highly recalcitrant to medical and surgical therapy [22]. El-Shazly et al. demonstated that NK cells infiltrated into the nasal epithelium in allergic CRS patients, but not in non-allergic CRS patients [23]. Thus, these studies suggest that NK cells may be involved in the chronic inflammation of CRS, particularly in eosinophilic inflammation; however, to date, studies on NK cell function in CRS patients have not yet been performed.

Given several studies showing the involvement of NK cells in airway disease, whose pathogenic features are similar to those of CRS, we hypothesized that the functions of NK cells from CRS patients may be altered. To address these questions, the NK cell functions of CRS patients were investigated.

## Methods

### Study subjects

Eighteen patients who had received endoscopic sinus surgery at least once for CRS were enrolled between October, 2003 and June, 2011 at the Asan Medical Center, Seoul. All patients fulfilled the established diagnostic criteria for CRS [1]. Generally, CRS that fails to respond to optimal medical and surgical therapy is diagnosed as recalcitrant CRS [24]. Further details on study subjects are in the supporting information section. In addition, clinical and demographic findings of patients are summarized in Tables S1 and S2. This study was approved by the Institutional Review Board of the Asan Medical Center (Seoul, Korea) and a signed written consent form was obtained before sample collection (approval numbers: 2011–0384).

### Isolation of peripheral blood cells

Peripheral blood mononuclear cells (PBMCs) were isolated from whole blood by density gradient centrifugation (LSM lymphocyte separation medium; MP Biomedicals) and assayed as detailed in the supporting information section.

### Antibodies used for flow cytometry

The anti-human mAbs used to determine NK-cell function and phenotype by flow cytometry are detailed in the supporting information section.

### Assay of NK cell degranulation

NK cell degranulation was determined by the cell surface expression of CD107a, as described (Dataset S1.) [25].

### Intracellular cytokine staining of NK cells

Nonadherent peripheral blood lymphocytes (PBLs) (2×10^5^ cells) were stimulated with an equal number of 721.221 (hereafter referred to as 221) [26] or K562 cells (American Type Culture Collection) for 1 hour at 37°C. Thereafter, brefeldin A (GolgiPlug; BD Bioscience) was added, followed by an additional 5 hours of incubation for a total of 6 hours. Details of the method for intracellular cytokine staining are in the supporting information section.

### Cytotoxicity assays

The cytotoxicity of primary NK cells against sensitive target cells was determined by a Europium-based cytotoxicity assay, as previously described [27]. PBLs served as the effector cells.

### Statistical analysis

All data were analyzed by using GraphPad Prism v.4.00 (GraphPad Software Inc). The groups were compared by using a nonparametric Mann-Whitney *U* test and Fisher's exact tests (two-tailed). Statistical significance was defined as *P*<0.05, and the degree of significance was presented as follows: *P<0.05; **P<0.005; ***P<0.001.

## Results

### NK cell functions in CRS patients are impaired

**Figure 1:**
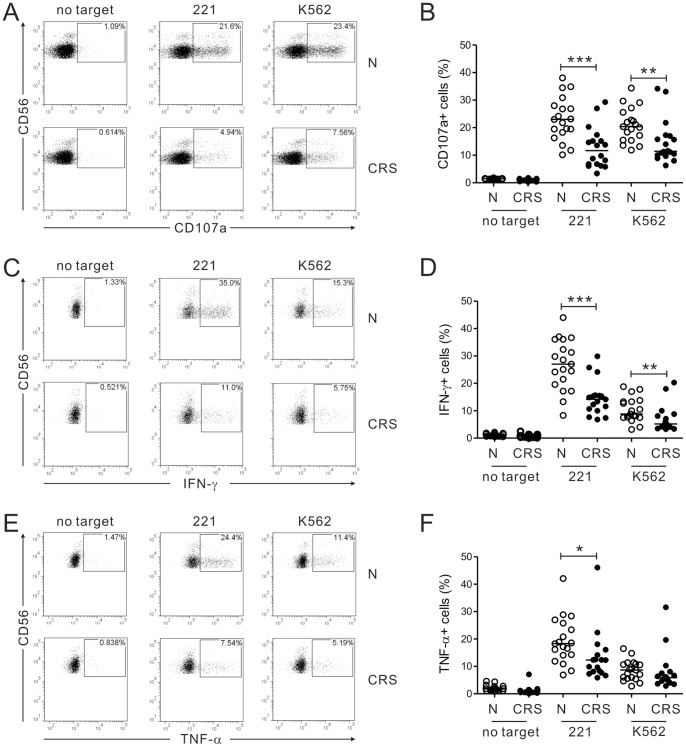
Patients with CRS have impaired NK cell functions. PBLs from the controls (N, *n* = 19) or patients with CRS (*n* = 18) were incubated with or without sensitive target cells. (A, B) Degranulation of NK cells, as measured by cell-surface expression of CD107a. (C-F) Cytokine production of NK cells, as measured by intracellular expression of IFN-γ (C, D) and TNF-α (E, F). (A, C, E) Representative FACS profiles showing the percentages of CD107a+ NK cells (A), IFN-γ+ NK cells (C) and TNF-α+ NK cells (E). (B, D, F) Summary graphs of the statistical dot plots showing the expression of CD107a (B), IFN-γ (D) or TNF-α (F) by NK cells. Horizontal bars indicate the medians. Statistical differences between the groups were evaluated with the nonparametric Mann-Whitney *U* test. *P<0.05; **P<0.005; ***P<0.001.

The CD3-CD56+NK cells in PBLs were identified by a FACS gating strategy (Fig. S1). CRS NK cells degranulated significantly poorly than control NK cells (*P*<0.001 against 221; *P*<0.005 against K562) (Figs. 1A,B). Since target-cell lysis by NK cells correlates with their degranulation efficiency [28], it was not surprising that NK cell cytotoxicity against 221 and K562 was also impaired in the CRS group (data not shown). CRS NK cells also produced significantly less IFN-γ against 221 and K562 (*P*<0.001 against 221; *P*<0.005 against K562) (Figs. 1C,D) and TNF-α against 221 (*P*<0.05) (Figs. 1E,F). Thus, NK cells of CRS patients were impaired in their ability to degranulate and produce IFN-γ and TNF-α. In control experiment, upon stimulation with PMA and ionomycin, both NK cells showed a similar degree of IFN-γ production (Fig. S2).

### NK cell dysfunction in CRS is associated with a poor clinical prognosis

**Figure 2:**
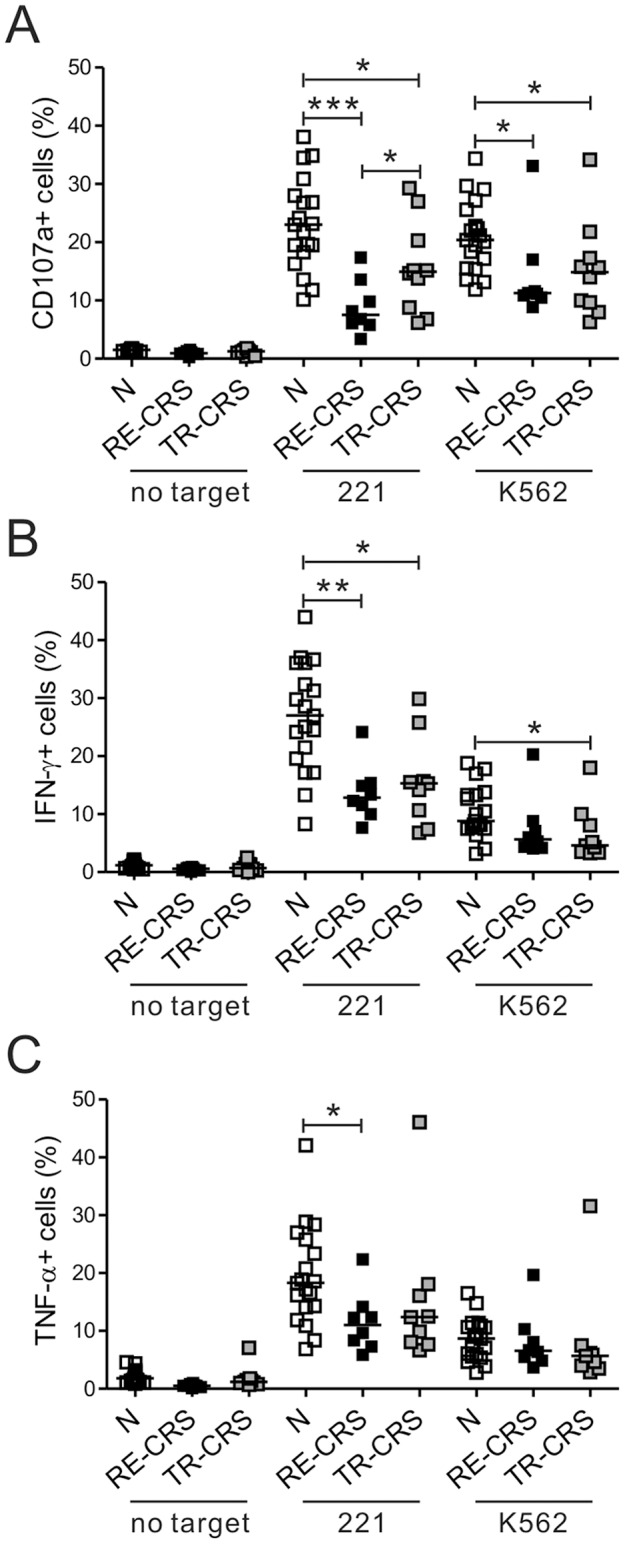
NK cell dysfunction associates with the clinical severity of CRS. The patients with CRS were divided into the treatment-responsive (TR-CRS) and recalcitrant (RE-CRS) groups on the basis of the clinical severity and responsiveness to medication. The PBLs from the controls (N, *n* = 19), the RE-CRS group (*n* = 8), and the TR-CRS group (*n* = 10) were incubated with or without the indicated target cells, as described in Figure 1. The three groups were compared via summary graphs of the statistical dot plots in terms of the CD107a (A), IFN-γ (B) or TNF-α (C) expression by NK cells. Horizontal bars denote the medians. *P<0.05; **P<0.005; ***P<0.001.

Eighteen CRS patients were divided on the basis of their long-term treatment outcomes into two subgroups, namely, the treatment-responsive group (n = 10) and the recalcitrant group (n = 8). Compared to the control group, NK cells from the recalcitrant group exhibited a marked impairment in NK cell degranulation after stimulation with 221 and K562 targets (*P*<0.001 against 221; *P*<0.05 against K562) (Fig. 2A). This impairment was more pronounced than the impairment in the treatment-responsive group (*P*<0.001 vs. *P*<0.05 against 221). Likewise, after stimulation with 221 cells, NK cells from the recalcitrant group produced much less IFN-γ (*P*<0.005) and TNF-α (*P*<0.05) than the control cells, while this decrease was less significant in the treatment-responsive group (*P*<0.005 vs. *P*<0.05 for IFN-γ production) (Figs. 2B,C). However, NK cell population in CRS patients was not significantly different from that in control group (Figs. S3,S4). Thus, the patients with recalcitrant CRS had the most severe defects in NK cell effector functions, suggesting that there is an association between clinical prognosis of CRS and NK cell dysfunction.

### Alterations in the NK cell-receptor expression in the CRS patients

**Figure 3:**
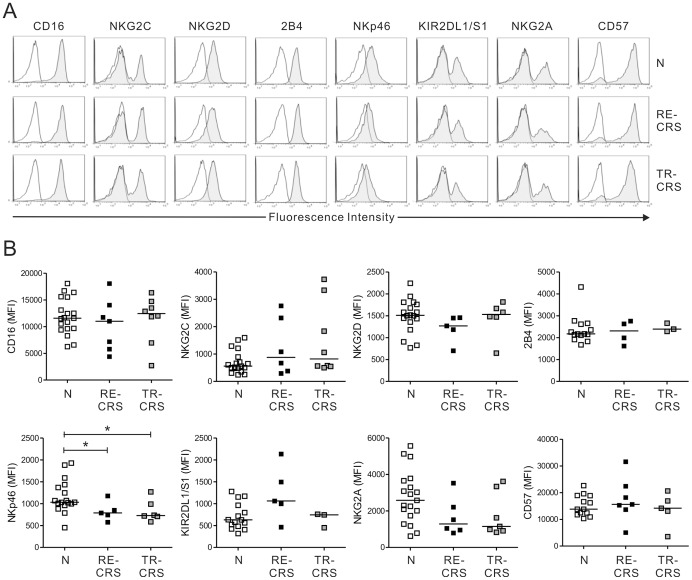
Patients with recalcitrant CRS have altered NK cell receptor expression patterns. (A) Representative FACS profiles showing the expression of the CD16, NKG2C, NKG2D, 2B4, NKp46, KIR2DL1/S1, NKG2A and CD57 receptors (shaded histogram) on gated NK cells in normal donors (N), patients with recalcitrant CRS (RE-CRS), and patients with treatment-resistant CRS (TR-CRS). Isotype control staining is shown as solid lines. (B) Summary graphs of statistical dot plots showing the mean fluorescence intensity (MFI) of the indicated receptor expression on NK cells of the different study groups. Horizontal bars indicate the medians. *P<0.05.

The current model of NK-cell activation and inhibition is based on a balance between signals from activating and inhibitory receptors [8]. Thus, the expression of activating and inhibitory receptors on NK cells from the CRS patients and the control subjects was assessed. The two groups did not differ significantly in terms of the expression of activating receptors, namely, CD16, NKG2C, NKG2D and 2B4 (Fig. S5). However, the CRS group had significantly lower level of NKp46 expression on NK cells. The two groups were also comparable in terms of their expression of the inhibitory receptor KIR2DL1/S1, and the expression of CD57, a marker of the terminal maturation of NK cells [29]. Analysis of the recalcitrant and treatment-responsive subgroups revealed that both NK cells expressed significantly less NKp46 than the control NK cells (*P*<0.05) (Fig. 3). The two subgroups and control group did not differ in terms of the expression of other NK-cell receptors.

### Asthma is an important risk factor for NK-cell dysfunction in CRS patients

**Figure 4:**
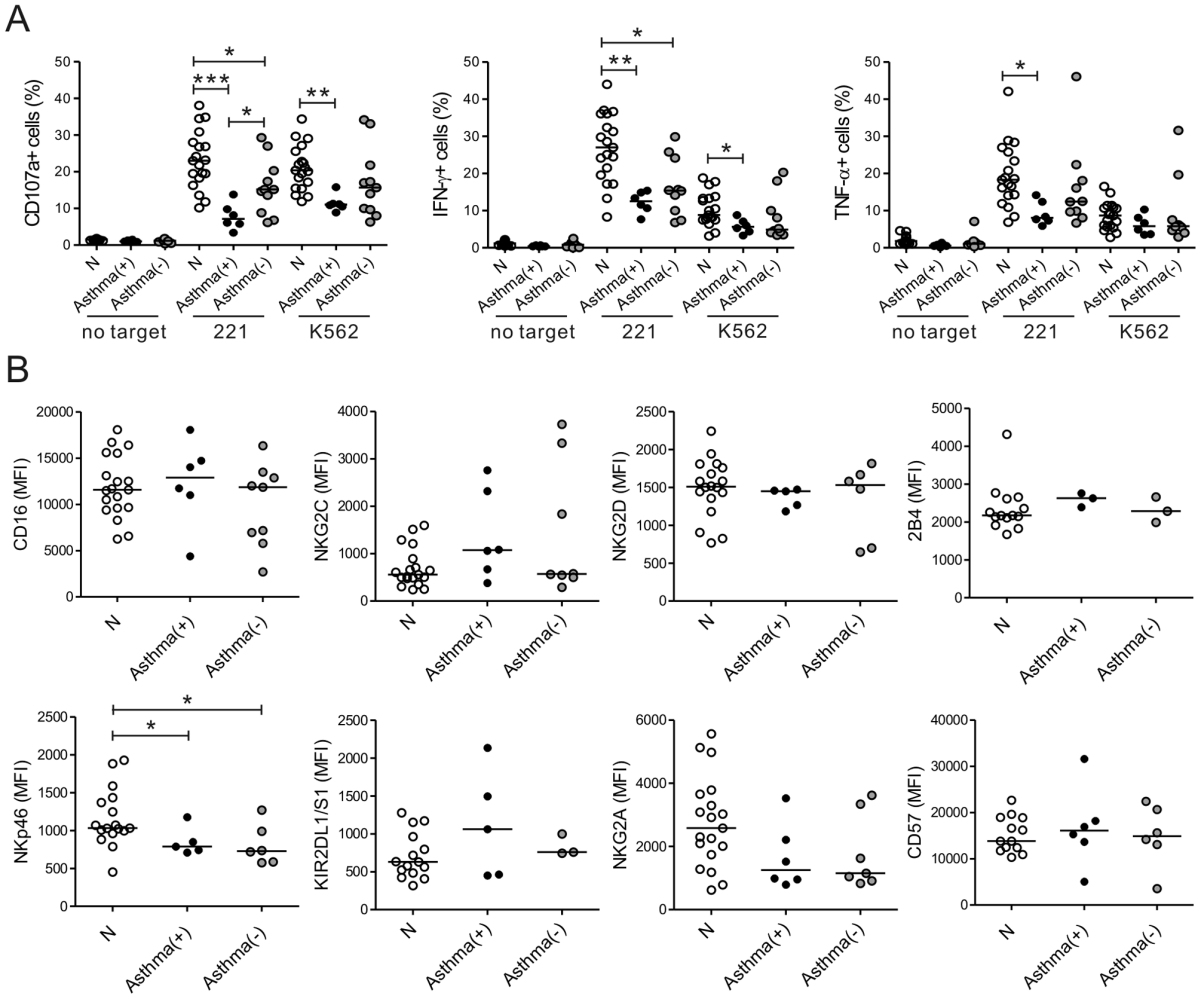
Asthma is an important risk factor for NK cell dysfunction in CRS and for CRS exacerbation. The patients with CRS were classified into the asthma (+) and asthma (−) groups based on the diagnosis of clinical symptoms of asthma. (A) PBLs from normal controls (N, *n* = 19), the asthma (+) patients (*n* = 7) and the asthma (−) patients (*n* = 11) were stimulated and analyzed, as described in Figure 2. The three groups were compared in terms of CD107a, IFN-γ or TNF-α expression on NK cells. (B) Summary graphs of statistical dot plots showing the MFI of the indicated receptor expression on NK cells between the groups. *P<0.05; **P<0.005; ***P<0.001.

Several reports suggest that NK cell activity in PBMCs from asthmatics is skewed towards a Th2-cytokine-producing phenotype that results in less IFN-γ production [14], [15]. Therefore, the association of asthma with function of NK cells from the CRS group was assessed by dividing the 18 patients into two study groups based on the concomitant presence of asthma. Seven patients were in the asthma (+) group and 11 were in the asthma (−) group. Compared to the control group, the asthma (+) group exhibited a severe impairment in NK-cell degranulation efficiency (*P*<0.001 against 221; *P*<0.005 against K562); however, this defect was less significant in the asthma (−) group (*P*<0.05 against 221) (Fig. 4A). As anticipated, the asthma (+) group also exhibited defective NK-cell production of IFN-γ (*P*<0.005 against 221; *P*<0.05 against K562) and TNF-α (*P*<0.05 against 221) relative to the control group; this difference was less pronounced in the asthma (−) group (*P*<0.005 vs. *P*<0.05 against 221 for IFN-γ production) (Fig. 4A). However, the asthma (+), asthma (−) and control groups did not differ in terms of the frequencies of NK cells and their subpopulations (Fig. S6). With regard to the expression of NK cell receptors, both asthma (+) and asthma (−) groups expressed significantly less NKp46 than the control group (*P*<0.05); there was no difference in terms of other receptors that were analyzed (Fig. 4B). Given that the majority of the patients with recalcitrant CRS were asthmatic (75%), these data suggest that asthma associates with CRS exacerbation and is an important risk factor for impaired NK-cell functions.

### NK cell functions in treatment-responsive CRS patients without concomitant asthma are also defective

**Figure 5:**
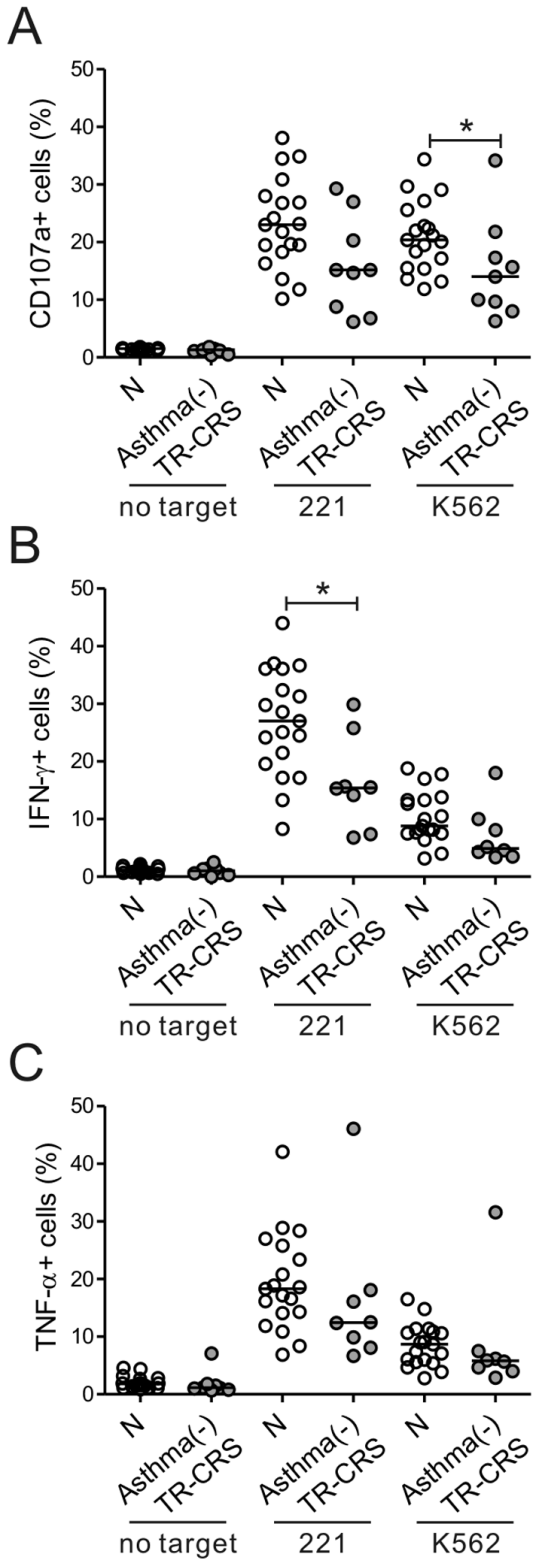
Treatment-responsive patients with CRS without asthma also have defective NK cell functions. The single asthmatic patient in the treatment-responsive (TR-CRS) group was excluded, thus yielding the asthma (−) TR-CRS group (*n* = 9). The PBLs from these patients and normal donors (N, *n* = 19) were stimulated and analyzed, as described in Figure 2. The two groups were compared in terms of the CD107a (A), IFN-γ (B) or TNF-α (C) expression by their NK cells. *P<0.05.

Since NK cell dysfunction was also observed in the asthma (−) group (Fig. 4A), it may be that asthma is not a prerequisite for impaired NK-cell function. To test this, the one patient with asthma in the treatment-responsive group was excluded. The remaining nine patients were then compared to the control subjects in terms of NK cell function. The NK cells from these patients had significantly decreased degranulation efficiency against K562 (*P*<0.05) (Fig. 5A). They also produced less IFN-γ and TNF-α after 221 cell stimulation; this decrease was significant for IFN-γ production (*P*<0.05) (Figs. 5B,C). Thus, treatment-responsive CRS patients who did not have concomitant asthma also had defective NK cell function. This suggests that asthma contributes to CRS exacerbation but is not the only determinant of NK cell dysfunction in CRS pathogenesis.

### Peripheral blood eosinophil counts are associated with impaired NK-cell functions in CRS

**Figure 6:**
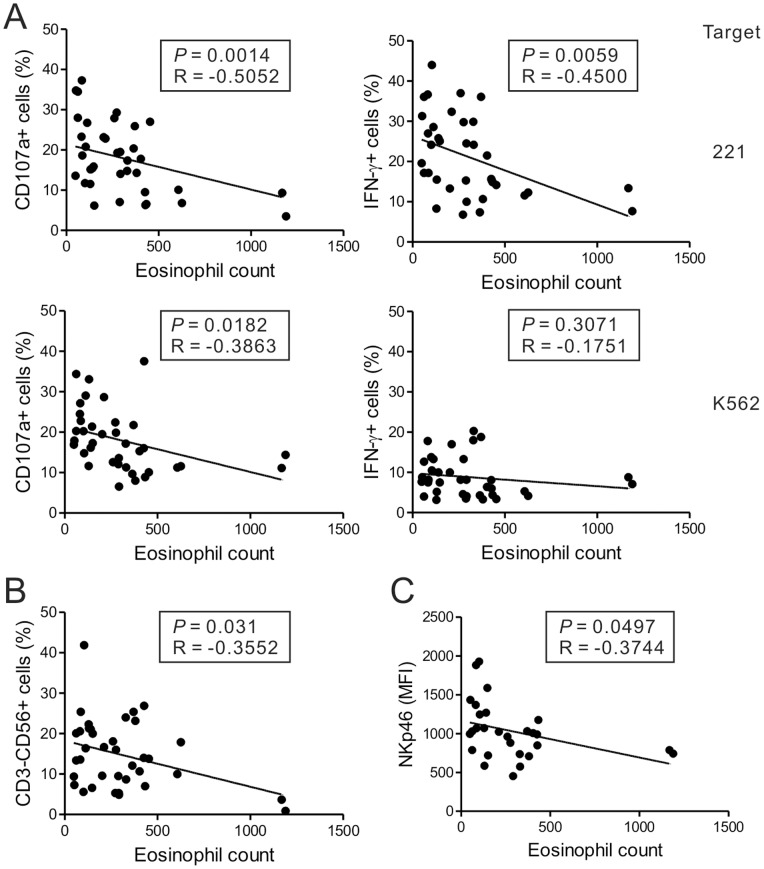
Peripheral blood eosinophil counts associate with impaired NK cell functions. (A) The eosinophil counts correlated negatively with the percentages of CD107a- or IFN-γ-positive NK cells after stimulation with 221 (top panel) or K562 (bottom panel) cells. (B) The eosinophil counts correlated inversely with the frequency of NK cells. (C) The eosinophil counts correlated inversely with the percentages of NKp46-expressing NK cells. Pearson's correlation coefficients (R) and *P* values are shown.

Since several studies have shown correlations between the numbers of peripheral and tissue eosinophils in CRS patients [22], the correlation between peripheral blood eosinophil counts and NK cell function was assessed to evaluate the relationship between NK cell function and eosinophilic inflammation. Interestingly, eosinophil counts correlated inversely with the frequencies of CD107a+ and IFN-γ+ NK cells after stimulation with 221 or K562 cells (Fig. 6A). There was also an inverse correlation between the eosinophil counts and the frequency of NK cells in the peripheral blood (Fig. 6B). The eosinophil counts also correlated inversely with the level of NKp46 expression on NK cells (Fig. 6C). These data suggest that peripheral blood eosinophil counts associate with the impairment of NK cell functions in CRS patients.

## Discussion

The disease CRS is marked by persistent mucosal inflammation [3]. It is possible that a combination of host susceptibility to microbial elements and defects in innate immunity are contributors to the chronic inflammatory state [4]. Previous studies of the innate immune system in CRS focused primarily on toll-like receptors (TLRs), B-cell activating factor of the TNF family, and epithelial function [30]. One previous study showed the increased migration of NK cells to the nasal mucosa of allergic CRS, suggesting a probable role of NK cells in the pathogenesis of CRS [23]. However, in our study, we investigated various NK cell functions in CRS patients and showed that these patients have functionally deficient NK cells.

Activated NK cells lyse target cells directly by exocytosis of cytotoxic granules; they also secrete cytokines such as IFN-γ and TNF-α that mediate their immune response to infection [31]. In this manner, NK cells function as an important sentinel of the immune system: not only do they serve as primary responder, they also alert the host to the presence of infectious organisms. In the present study, *in vitro* activation of NK cells from CRS patients revealed that their degranulation efficiency was impaired and their production of IFN-γ and TNF-α was reduced. Similarly, other studies showed that NK cell function is also impaired in pulmonary tuberculosis and in COPD [10]–[13]. In addition, in mouse models of pulmonary fibrosis, the lack of NK cell recruitment resulted in the absence of IFN-γ in the lung and enhanced fibrosis; exogenous IFN-γ treatment was therapeutic [32], [33], demonstrating the importance of NK cell-derived IFN-γ in regulating pulmonary fibrosis. These findings together suggest that NK cells are protective against respiratory infections and inflammatory disorders of the lung. Considering that the nasal cavity is often the first point of contact between the airway mucosa and external organisms, it is possible to deduce that the impaired NK cell effector functions in CRS patients may be an important etiological factor that contributes to the persistence of the sinonasal infection and inflammation.

In a small subset of CRS patients, various combinations of medical and surgical treatments fail. The disease in these patients is therefore often termed recalcitrant CRS. The numerous studies have sought to determine how the innate immune system contributes to the pathogenesis [34], [35]. A recent study revealed that recalcitrant CRS patients had significantly decreased expression of TLR2 and TLR9 compared to treatment-responsive patients [34]. A study of treatment-resistant CRS patients found evidence of impaired regulatory mechanisms for IFN-γ production in sinus lavage cells and decreased IFN-γ production by PBMCs [35]. Our present study also showed that the patients with recalcitrant CRS had more defects in NK cell-effector functions including IFN-γ production than the treatment-responsive CRS group and the non-CRS controls. This fact suggests that there is a relationship between the clinical prognosis of CRS and NK cell dysfunction.

Of interest is the finding that K562 and 221 target cells showed differential susceptibility to cytotoxic degranulation and cytokine production by NK cells from the recalcitrant group. Compared to the control group, defective effector functions of NK cells from the recalcitrant group were more pronounced when 221 rather than K562 cells were used as target cells. K562 cells are of the erythroleukemia type and known to express ligands for NKG2D, DNAM-1, and NKp30 receptors [36], [37]. 221 cells are EBV-transformed B cell line, and their lysis by NK cells is associated with 2B4, NKp44, and NKp46 receptors [38], [39]. Although not fully characterized the ligands for NK cell receptors on K562 and 221 cells, we speculate that the different receptor –ligand interaction between NK cells and target cells might contribute to the differential susceptibility of K562 and 221 cells to NK cells from the recalcitrant group. In this respect, further studies are warranted to evaluate effector functions of CRS NK cells in the context of specific receptor –ligand interaction.

It has long been known that there is an association between CRS and asthma, especially severe asthma. Patients with severe steroid-dependent asthma have significantly more sinonasal involvement than patients with mild to moderate asthma [40]. Furthermore, NK cell activity in patients with asthma is skewed toward a Th2-cytokine-producing phenotype and is associated with diminished IFN-γ production [14], [15]. In the present study, the CRS group with asthma showed severe impairment of NK cell degranulation efficiency and IFN-γ and TNF-α production, whereas these defects were less pronounced in the CRS group without asthma. This result suggests that asthma is associated with CRS exacerbation, and is an important risk factor for impaired NK cell functions. Since the CRS group without asthma also exhibited a certain degree of NK cell dysfunction, the treatment-responsive group was examined more closely by excluding the single person with asthma. The resulting treatment-responsive-without-asthma group showed significantly reduced degranulation efficiency and IFN-γ production relative to the controls. This suggests that while asthma contributes to CRS exacerbation and impaired NK cell effector function, it is not the only determinant of NK-cell dysfunction in CRS pathogenesis.

One of the interesting findings in the present study was that the peripheral blood eosinophil counts correlated inversely with the NK cell effector functions, namely degranulation efficiency and IFN-γ production. Eosinophilic CRS is associated with asthma and peripheral blood eosinophilia [20] and is highly recalcitrant to medical and surgical therapy [21]. There is evidence that NK cells help regulate eosinophilic inflammation in several airway diseases [18], [19], [41]. With regard to the pathogenesis of CRS, it has been reported that eosinophilic CRS with nasal polyp is associated with exaggerated Th1/Th2/Th17 mixed responses compared to non-eosinophilic CRS with nasal polyp [5]; however, the underlying pathogenic mechanism of eosinophilic inflammation in CRS is still unclear. Given the results of previous studies in other airway diseases, it is possible that the pathogenic pathway of eosinophilic inflammation is related to the impaired function of NK cells in CRS. Supporting this hypothesis is the fact that the recalcitrant CRS group, which had the most severe defects in NK cell function, had significantly higher eosinophil counts than the other groups (Tables S1,S2). Further studies are needed to investigate how NK cell dysfunction contributes to the eosinophilic inflammation in CRS pathogenesis.

A limitation of our study is that we analyzed NK cells and eosinophils in the peripheral blood and not from the nasal samples. However, as a follow-up study, the role of NK cells in CRS pathogenesis has been examined using a mouse model of CRS. This study has revealed that mice that develop CRS exhibited impaired NK cell effector functions and increased eosinophilia in peripheral blood as well as in sinonasal tissues (Kim et al., unpublished data), thus supporting a correlation of NK cell function with eosinophilic inflammation. Given that CRS is marked by chronic inflammatory state, it is possible to postulate that NK cell dysfunction in CRS patients is attributed in part to functional exhaustion of NK cells by a chronic trigger. Previous studies have revealed a progressive loss of viral-specific CD8+ T cell functions during chronic viral infections and a crucial role for PD-1 expression in such cellular exhaustion. However, we observed a low and comparable level of PD-1 expression on NK cells between control and CRS groups (data not shown). Moreover, it remains unclear the expression and role of PD-1 in functional exhaustion of NK cells during chronic inflammatory diseases such as CRS. Thus, further study is required to assess the contribution of other regulatory mechanism(s) including NK cell exhaustion as potential mechanisms that accounts for NK cell dysfunction in CRS.

In summary, the present study showed that CRS patients have impaired NK cell effector functions, including cytotoxic degranulation and IFN-γ and TNF-α production. Moreover, these NK cell dysfunctions were correlated with a deteriorated clinical course. These impairments were associated with poor prognostic factors such as concomitant asthma and peripheral blood eosinophilia. These observations reinforce the notion that NK cells may play an important role in regulating the inflammatory process in CRS pathogenesis. The present study also suggests that, besides concomitant asthma and peripheral blood eosinophilia, NK cell function may be useful as a prognostic indicator for CRS patients.

## Supporting Information

Figure S1 FACS gating strategy.
(DOCX)

Figure S2 Patients with CRS have comparable degree of IFN-γ production upon stimulation with PMA and ionomycin.
(DOCX)

Figure S3 Patients with CRS have comparable NK-cell frequencies.
(DOCX)

Figure S4 Patients with CRS are comparable to normal controls in terms of the distribution of individual NK cell subsets.
(DOCX)

Figure S5 Patients with CRS have reduced percentages of NKp46-expressing NK cells.
(DOCX)

Figure S6 Patients with CRS with or without asthma are comparable to the controls in terms of NK cell frequency.
(DOCX)

Dataset S1 Supporting Methods.
(DOCX)

Table S1 Clinical and demographic data of the patients with CRS and the control subjects.
(DOCX)

Table S2 Comparison of the treatment-responsive and recalcitrant CRS groups in terms of clinical features.
(DOCX)
