# Supplementary material for: Natural Killer Cells from Patients with Chronic Rhinosinusitis Have Impaired Effector Functions
Source: PLoS One. 2013 Oct 18;8(10):e77177. doi: 10.1371/journal.pone.0077177 (PMC3799692; doi:10.1371/journal.pone.0077177)

**Figure S1.** FACS gating strategy. Profiles showing the gating strategy for identifying the CD3-CD56+ NK cells within the lymphocyte gate. Gating strategy: forward scatter (FSC) *vs*. side scatter (SSC) (left panel), then FSC-Height *vs*. FSC-Area (middle panel), and CD3 *vs*. CD56 (right panel).


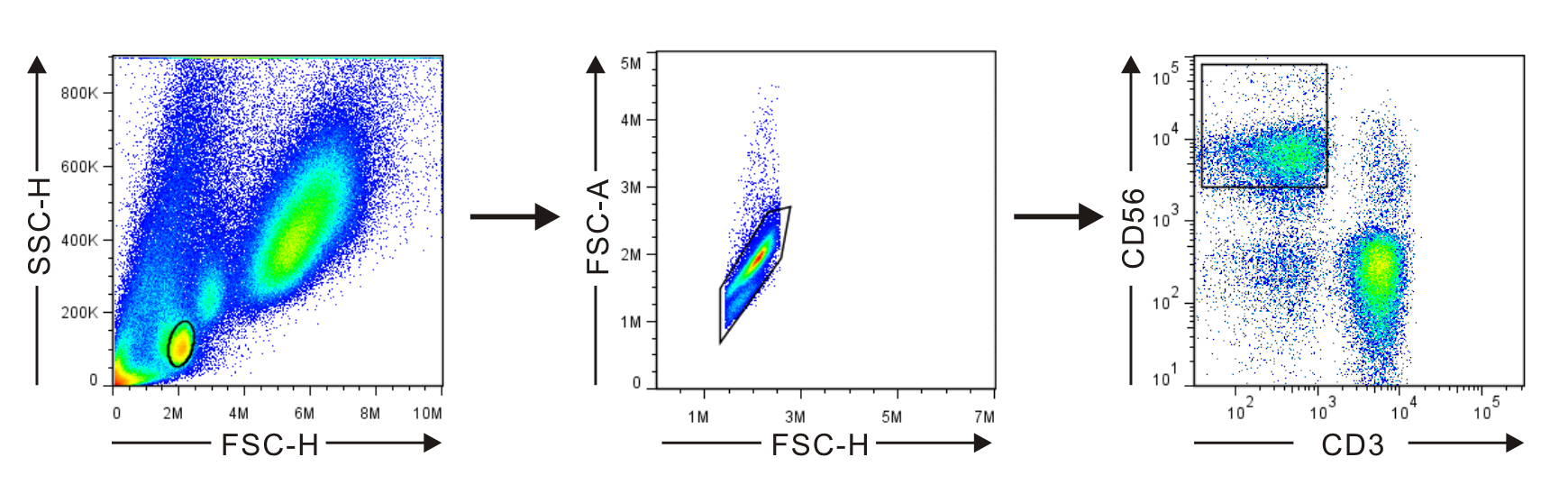

Supplement: Figure S1 — FACS gating strategy. (DOCX) [file pone.0077177.s001.docx]
