# Supplementary material for: Natural Killer Cells from Patients with Chronic Rhinosinusitis Have Impaired Effector Functions
Source: PLoS One. 2013 Oct 18;8(10):e77177. doi: 10.1371/journal.pone.0077177 (PMC3799692; doi:10.1371/journal.pone.0077177)

**Figure S2.** Patients with CRS have comparable degree of IFN-γ production upon stimulation with PMA and ionomycin. Comparison of the controls and the patients with CRS in terms of IFN-γ+ NK cells in the PBLs.


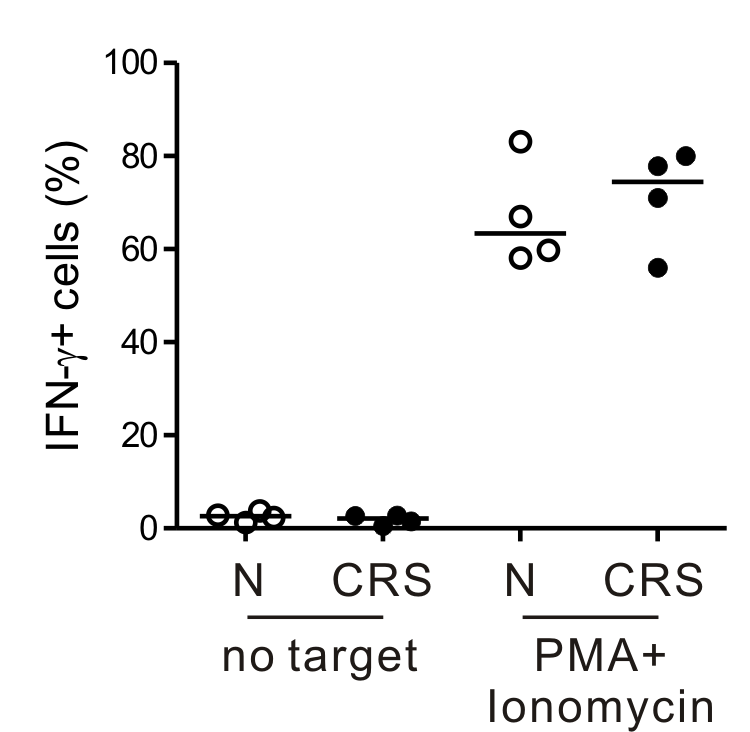

Supplement: Figure S2 — Patients with CRS have comparable degree of IFN-γ production upon stimulation with PMA and ionomycin. (DOCX) [file pone.0077177.s002.docx]
