# Supplementary material for: Natural Killer Cells from Patients with Chronic Rhinosinusitis Have Impaired Effector Functions
Source: PLoS One. 2013 Oct 18;8(10):e77177. doi: 10.1371/journal.pone.0077177 (PMC3799692; doi:10.1371/journal.pone.0077177)

**Figure S3.** Patients with CRS have comparable NK-cell frequencies. (A) The percentages of total NK cells in the PBMCs from normal controls (N, *n*=19) or patients with CRS (*n*=18). (B) Comparison of the controls, the recalcitrant CRS group (RE-CRS, *n*=8), and the treatment-responsive CRS group (TR-CRS, *n*=10) in terms of total NK cells in the PBMCs.


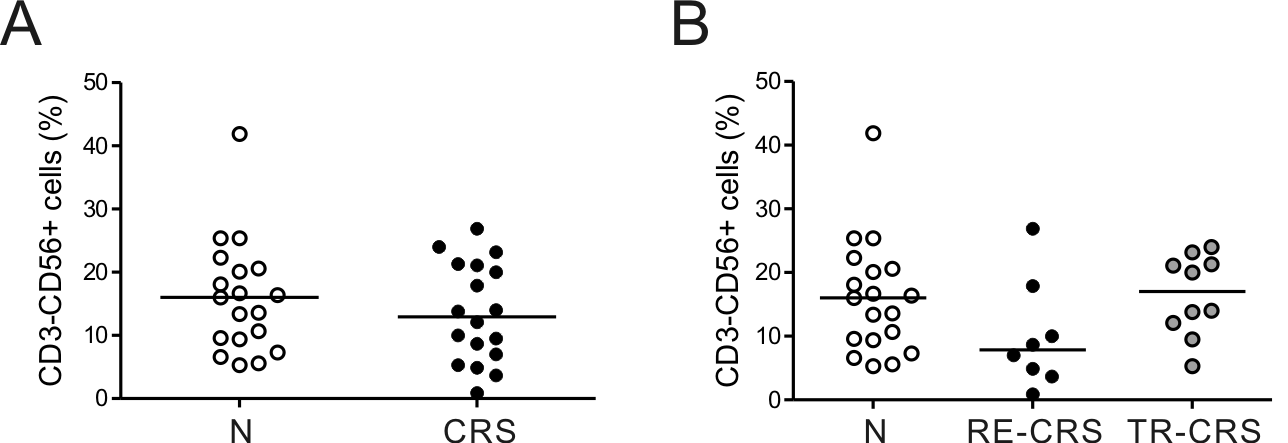

Supplement: Figure S3 — Patients with CRS have comparable NK-cell frequencies. (DOCX) [file pone.0077177.s003.docx]
