# Supplementary material for: Natural Killer Cells from Patients with Chronic Rhinosinusitis Have Impaired Effector Functions
Source: PLoS One. 2013 Oct 18;8(10):e77177. doi: 10.1371/journal.pone.0077177 (PMC3799692; doi:10.1371/journal.pone.0077177)

**Figure S4.** Patients with CRS are comparable to normal controls in terms of the distribution of individual NK cell subsets. (A, B) Comparison of the patients with CRS (A) or the RE-CRS and TR-CRS subgroups (B) to the normal controls in terms of the distribution of different NK-cell subsets (CD56^dim^CD16+, CD56^bright^CD16+ and CD56^bright^CD16-) in the total NK-cell fraction.


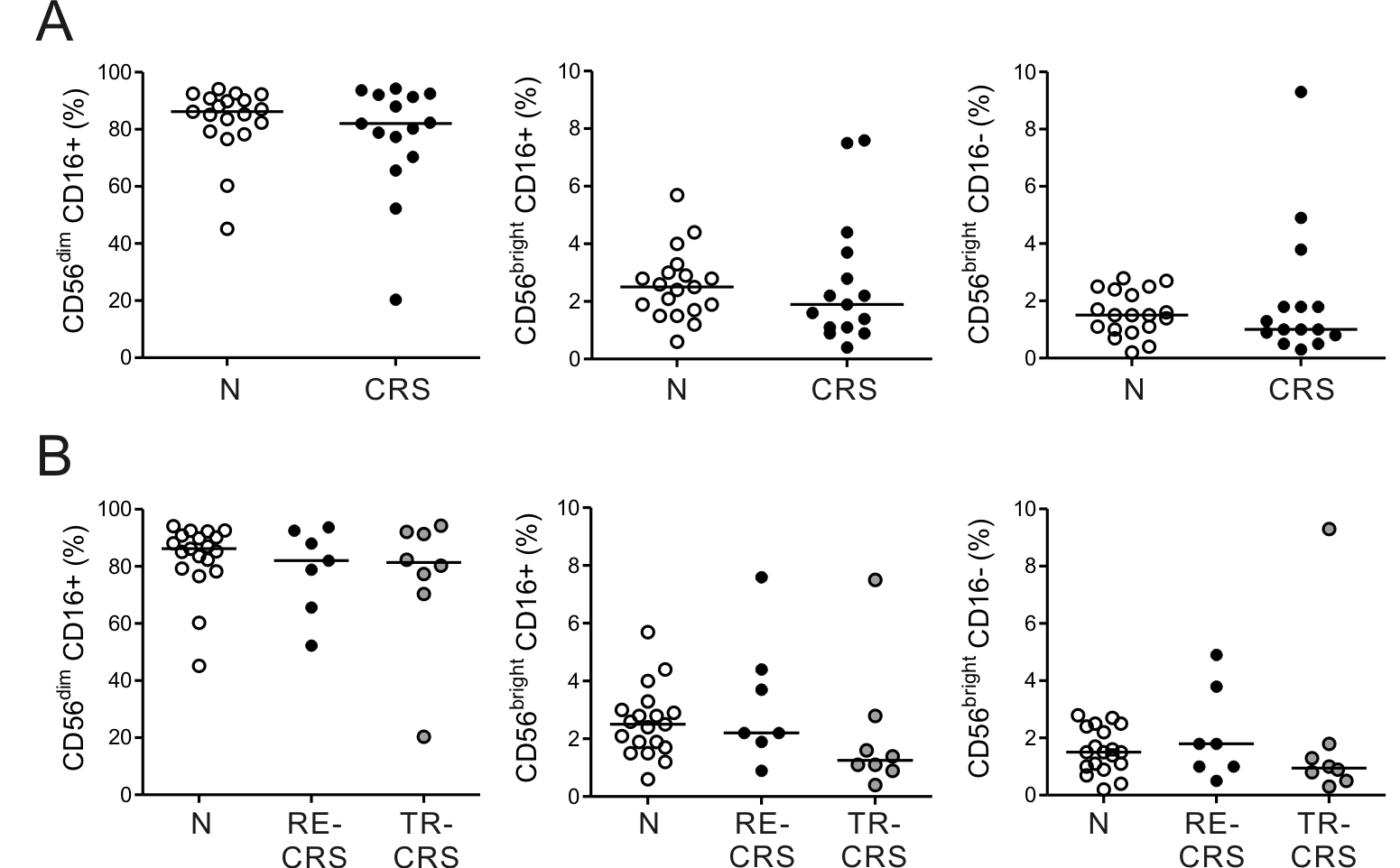

Supplement: Figure S4 — Patients with CRS are comparable to normal controls in terms of the distribution of individual NK cell subsets. (DOCX) [file pone.0077177.s004.docx]
