# Supplementary material for: Natural Killer Cells from Patients with Chronic Rhinosinusitis Have Impaired Effector Functions
Source: PLoS One. 2013 Oct 18;8(10):e77177. doi: 10.1371/journal.pone.0077177 (PMC3799692; doi:10.1371/journal.pone.0077177)

**Figure S5.** Patients with CRS have reduced percentages of NKp46-expressing NK cells. Summary graphs of statistical dot plots with the medians (horizontal bars) showing the MFI of the indicated receptor expression on NK cells in the controls or patients with CRS. The groups were compared statistically by using the Mann-Whitney *U* test. **P*<0.05.


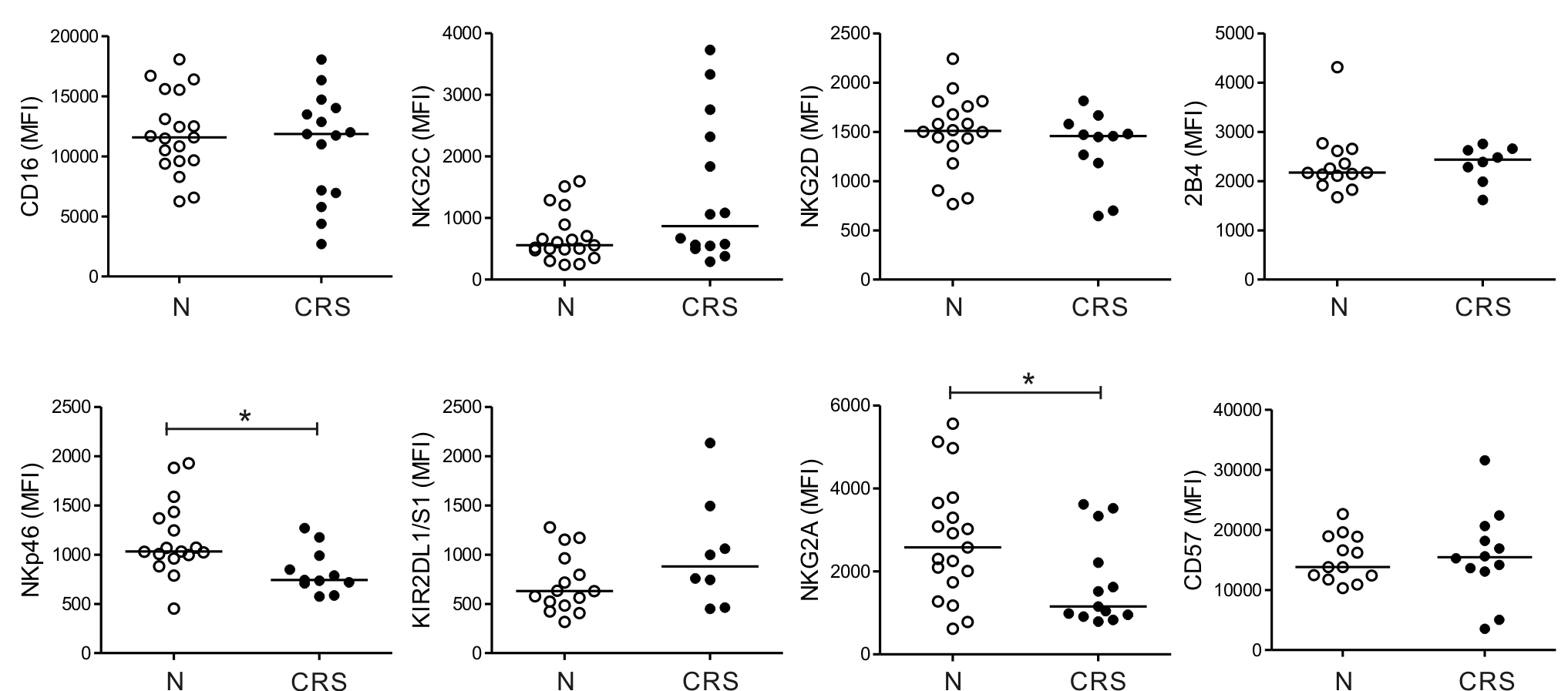

Supplement: Figure S5 — Patients with CRS have reduced percentages of NKp46-expressing NK cells. (DOCX) [file pone.0077177.s005.docx]
