# Supplementary material for: Natural Killer Cells from Patients with Chronic Rhinosinusitis Have Impaired Effector Functions
Source: PLoS One. 2013 Oct 18;8(10):e77177. doi: 10.1371/journal.pone.0077177 (PMC3799692; doi:10.1371/journal.pone.0077177)

**Figure S6.** Patients with CRS with or without asthma are comparable to the controls in terms of NK cell frequency. (A) Comparison of normal controls or patients with CRS who do or do not have asthma in terms of total NK cell numbers in PBMCs. (B) The three groups were compared in terms of the distributions of different NK cell subsets (CD56^dim^CD16+, CD56^bright^CD16+ and CD56^bright^CD16-) in the total NK cell fraction.


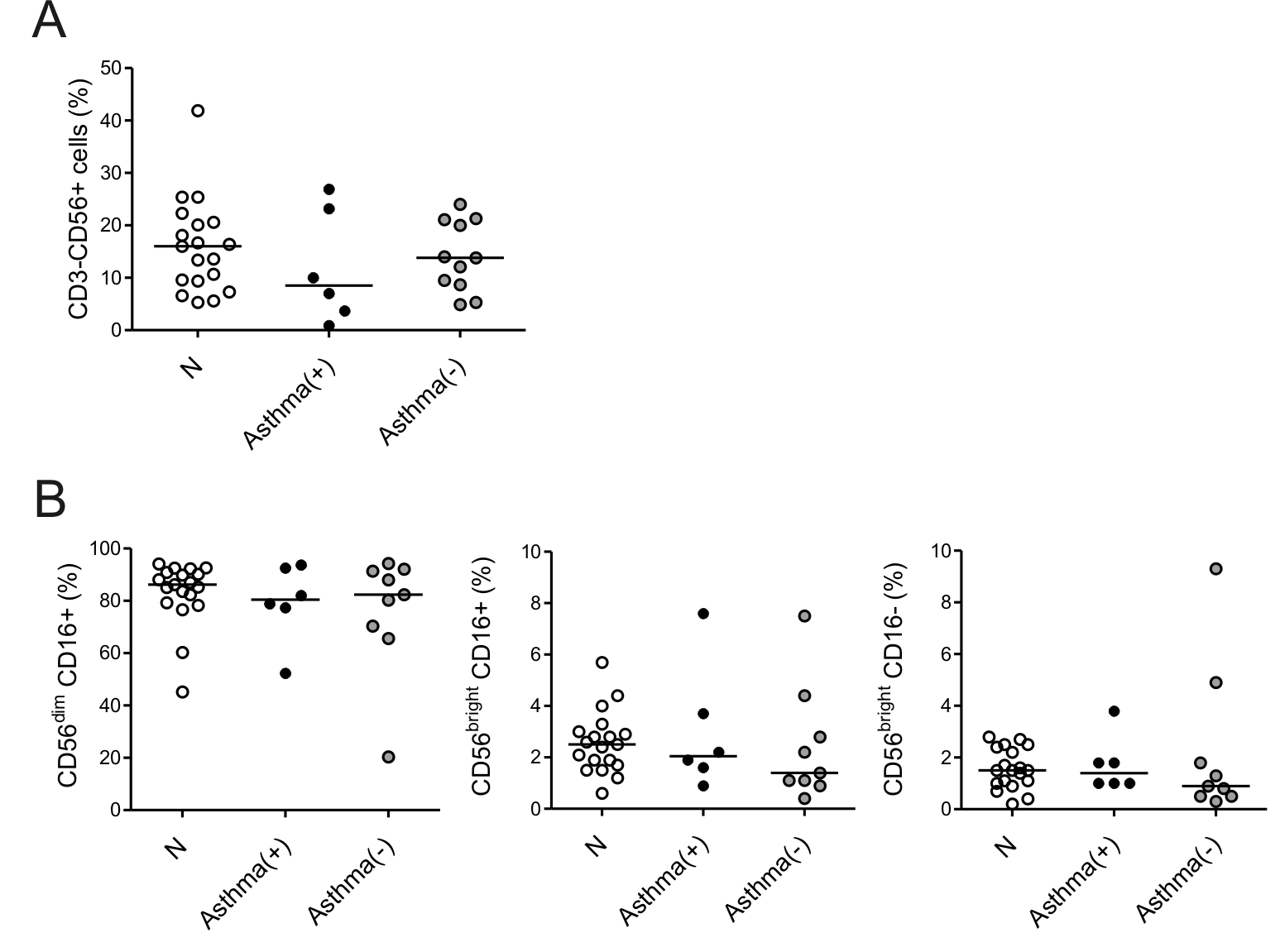

Supplement: Figure S6 — Patients with CRS with or without asthma are comparable to the controls in terms of NK cell frequency. (DOCX) [file pone.0077177.s006.docx]
