# Supplementary material for: Natural Killer Cells from Patients with Chronic Rhinosinusitis Have Impaired Effector Functions
Source: PLoS One. 2013 Oct 18;8(10):e77177. doi: 10.1371/journal.pone.0077177 (PMC3799692; doi:10.1371/journal.pone.0077177)
Supplement: Dataset S1 — Supporting Methods. (DOCX) [file pone.0077177.s007.docx]

**Dataset S1.** Supporting Methods

**Study subjects**

Eighteen patients who had received endoscopic sinus surgery at least once for CRS were enrolled between October, 2003 and June, 2011 at the Asan Medical Center, Seoul. All patients fulfilled the established diagnostic criteria for CRS (28). Clinical and demographic findings of patients are summarized in Tables S1 and S2. Patients with fungal sinusitis, current immunotherapy, aspirin desensitization, or cystic fibrosis were excluded. A further reason for exclusion was treatment with local or oral steroids within the previous 4 weeks. The controls were 19 volunteers who required planned surgery for a deviated nasal septum. None of the controls had experienced CRS and the presence of sinusitis was ruled out based on patients’ history, physical examination, plain sinus X-ray, and/or computed tomography (CT) imaging. The clinical and demographic data of both groups are shown in Table S1. Peripheral blood samples for testing NK cell functions and measuring eosinophil numbers were collected from each patient before surgery in the operating room. A complete peripheral blood cell count was obtained by automated analysis, and the percentage and absolute eosinophil count were calculated. This study was approved by the Institutional Review Board of the Asan Medical Center (Seoul, Korea) and a signed written consent form was obtained before sample collection.

**Classification of patients with CRS**

All patients with CRS were followed up for a minimum period of 14 months, with a mean follow up period of 36.3 months. Generally, CRS that fails to respond to optimal medical and surgical therapy is diagnosed as recalcitrant CRS (22). After a careful review of the past history and clinical course of the patients after sinus surgery, eight patients who continued to suffer from recurrent exacerbations of CRS after sinus surgery and did not respond to further medical therapy were placed in the recalcitrant CRS group (RE-CRS). The ten patients who responded to optimal medical and surgical therapy were included in the treatment-responsive CRS group (TR-CRS). Nasal polyps were found in five RE-CRS group patients (62.5%) and in six TR-CRS group patients (60.0%) but this difference was not statistically significant (*P=*0.314). However, Table S2 shows that the two groups differed significantly in terms of their clinical features and eosinophil counts.

**Isolation of peripheral blood cells**

Peripheral blood mononuclear cells (PBMCs) were isolated from whole blood by density gradient centrifugation (LSM lymphocyte separation medium; MP Biomedicals) and then cryopreserved. The frozen PBMCs were thawed later and suspended in complete RPMI medium [RPMI-1640 medium supplemented with 10% fetal bovine serum (FBS), 2 mM L-glutamine, 100 U/ml penicillin, and 100 μg/ml streptomycin] in the presence of 50 U/ml DNase I (Roche) to remove the DNA released by the dead cells and to prevent PBMC clumping. The cells were washed twice with complete RPMI medium, resuspended in the same medium at approximately 4×10^6^ cells/ml, and rested overnight at 37°C in a CO_2_ incubator.

**Antibodies used for flow cytometry**

The following anti-human mAbs were used to determine NK-cell function and phenotype by flow cytometry: anti-CD3-PerCP (clone SK7, BD Bioscience), anti-CD56-PE (clone NCAM16.2, BD Bioscience), anti-CD107a-FITC (clone H4A3, BD Bioscience), anti-IFN-γ-FITC (clone 25723.11, BD Bioscience), and anti-TNF (tumor necrosis factor)-α-FITC (clone MAb11, eBioscience). The following mAbs were used for phenotypic analysis of NK cells: anti-CD3-PerCP (clone SK7, BD Bioscience), anti-CD56-FITC (clone NCAM16.2, BD Bioscience), anti-CD16-PE (clone 3G8, BD Bioscience), anti-NKG2C-PE (clone 134591, R&D Systems), anti-NKG2D-PE (clone 149810, R&D Systems), anti-2B4-PE (CD244; clone C1.7, BD Bioscience), anti-NKp46-PE (CD335; clone 9E2, BD Bioscience), anti-KIR2DL1/S1-PE (CD158a; clone EB6, Beckman Coulter), anti-NKG2A-PE (CD159a; clone Z199, Beckman Coulter) and anti-CD57-PE (clone NK-1, BD Bioscience).

**Assay of NK cell degranulation**

NK cell degranulation was determined by the cell surface expression of CD107a, as described (23, 24). Briefly, nonadherent peripheral blood lymphocytes (PBLs) (2×10^5^ cells) were isolated after the plastic adherence of PBMCs, mixed with an equal number of K562 cells or 721.221 cells (hereafter referred to as 221), spun down for 3 min at 30×g, incubated for 2 hours at 37°C, and spun down again. The cell pellets were resuspended in FACS buffer (PBS with 2% FBS) and stained with anti-CD3-PerCP, anti-CD56-PE and anti-CD107a-FITC mAbs for 30 min in the dark at 4°C. Lymphocytes were gated on forward scatter/side scatter, and the CD107a expression of the CD3-CD56+ NK cells was analyzed by flow cytometry (BD Bioscience) and FlowJo software (Tree Star).

**Intracellular cytokine staining of NK cells**

PBLs (2×10^5^ cells) were stimulated with an equal number of K562 or 221 cells for 1 hour at 37°C. Thereafter, brefeldin A (GolgiPlug; BD Bioscience) was added, followed by an additional 5 hours of incubation for a total of 6 hours. The cells were then stained for surface markers with anti-CD3-PerCP and anti-CD56-PE mAbs for 30 min in the dark at 4°C. After washing the PBLs twice with FACS buffer, they were incubated in BD Cytofix/Cytoperm solution (BD Bioscience) for 20 min in the dark at 4°C. Before and after intracellular staining with anti-IFN-γ-FITC or anti-TNF-α-FITC mAb for 30 min in the dark at 4°C, the cells were washed twice with BD Perm/Wash buffer (BD Bioscience). The cells were then analyzed by flow cytometry gated on CD3-CD56+ NK cells.
