# Supplementary material for: Natural Killer Cells from Patients with Chronic Rhinosinusitis Have Impaired Effector Functions
Source: PLoS One. 2013 Oct 18;8(10):e77177. doi: 10.1371/journal.pone.0077177 (PMC3799692; doi:10.1371/journal.pone.0077177)
Supplement: Table S1 — Clinical and demographic data of the patients with CRS and the control subjects. (DOCX) [file pone.0077177.s008.docx]

**Table S1.** Clinical and demographic data of the patients with CRS and the control subjects.

|  | **CRS** | **Control subjects** |
| --- | --- | --- |
| Number of patient | 18 | 19 |
| Mean age (Range) | 44.5 (19 - 67) | 31.1 (16 - 63) |
| Gender (Male/Female) | 14/4 | 17/2 |
| Asthma (%) | 7 (38.9%) | 0 |
| Mean blood eosinophil count(count/μl) | 444 | 162 |
