# Supplementary material for: Natural Killer Cells from Patients with Chronic Rhinosinusitis Have Impaired Effector Functions
Source: PLoS One. 2013 Oct 18;8(10):e77177. doi: 10.1371/journal.pone.0077177 (PMC3799692; doi:10.1371/journal.pone.0077177)
Supplement: Table S2 — Comparison of the treatment-responsive and recalcitrant CRS groups in terms of clinical features. (DOCX) [file pone.0077177.s009.docx]

**Table S2.** Comparison of the treatment-responsive and recalcitrant CRS groups in terms of clinical features.

| **Groups** | **Recalcitrant group**  **(RE-CRS)** | **Treatment responsive group (TR-CRS)** | ***P* - value** |
| --- | --- | --- | --- |
| Number of patient | 8 | 10 |  |
| Mean age | 42.3 | 46.3 | 0.569 |
| Gender (Male/Female) | 6/2 | 8/2 | 0.800 |
| Duration of follow-up period (months) | 38.9 | 31.1 | 0.665 |
| Nasal polyps | 5 (62.5%) | 6 (60.0%) | 0.314 |
| Asthma | 6 (75.0%) | 1 (10.0%) | 0.013 |
| Lund-Mackay CT score | 17.9 | 11.0 | 0.026 |
| Duration of antibiotics medication (months/year) | 3.54 | 1.29 | 0.011 |
| Duration of oral steroid medication (months/year) | 1.72 | 0.39 | 0.002 |
| Blood eosinophil count  (count/μl) | 633 | 292.7 | 0.031 |
